# The effect of antidepressant treatment on white matter integrity in Major Depression

**DOI:** 10.1192/j.eurpsy.2022.555

**Published:** 2022-09-01

**Authors:** S. Tourjman, S. Potvin, R. Milan, E. Kouassi, D. Luck

**Affiliations:** 1 Institut Universitaire en Santé Mentale de Montréal, Psychiatry, Montréal, Canada; 2 McGill University, Medicine, Montréal, Canada

**Keywords:** Neuroimaging, Depression, antidepressant, DTI

## Abstract

**Introduction:**

White matter abnormalities have been identified in major depressive disorder (MDD). Although several diffusion tensor imaging studies found decreased fractional anisotropy (FA) in MDD, the effect of antidepressants (AD) treatment on white matter integrity has been insufficiently studied.

**Objectives:**

We sought to examine the effect of AD treatment of MDD on white matter, using DTI, in responders compared to nonresponders.

**Methods:**

We included 25 individuals with MDD (HAMD >/=20) without inflammatory, unstable medical/neurological conditions or prolonged duration (> 1 year),or AD or anti-inflammatory treatment >/=1 week preceding first evaluation. Evaluation before treatment and at 16 weeks included depression rating scales, a cognitive battery, inflammatory markers and MRI. Desvenlafaxine was initiated at 50mg with a possible increase to 100mg at 8 weeks.

**Results:**

Changes included: increased volume in responders in the right Inferior Fronto-Occipital fasciculus (p=0.0315) and Superior Longitudinal Fasciculus part 3 (p=0.0050); in remitters in the right Inferior Fronto-Occipital fasciculus (p=0.0359) and Superior Longitudinal Fasciculus part 2 (p<0.05) and 3 (p=0.0481); decreased volume in responders in the left Superior Longitudinal Fasciculus part 1 (p=0.0147) and left Corona Radiata(p<0.05); and in remitters in the left Superior Longitudinal Fasciculus part 1 (p=0.0109) and the Corpus Callosum part 5 (p<0.05); decreased FA in the right Cortico Spinal Tract in remitters (p=0.0175) and responders (p=0.0272), and an increase in FA in the left Uncinate Fasciculus in nonremitters (p=0.0493). These results lose significance following Bonferroni correction.

**Conclusions:**

Overall, AD treatment of MDD was not associated with significant changes in FA, whole brain, or specific tract volume in this study.

**Disclosure:**

This research was funded by Pfizer Canada.

